# Pan-cancer gene signature analysis of macrophage polarization and compound prediction for reprogramming tumor-associated macrophages toward M1-like macrophages^☆^

**DOI:** 10.1016/j.cpt.2025.05.002

**Published:** 2025-05-20

**Authors:** Xiaojing Liu, Cheng Liu, Yuting Jin, Jing Xu, Chunyan Xu, Wei Zhu

**Affiliations:** a Department of Oncology, The First Affiliated Hospital of Nanjing Medical University, Nanjing, Jiangsu 210029, China; b Department of Radiotherapy, The Friendship Hospital of Ili Kazak Autonomous Prefecture, Ili, Xinjiang 835000, China; c Department of Gastroenterology, Nanjing Drum Tower Hospital Clinical College of Nanjing Medical University, Nanjing, Jiangsu 210008, China; d Department of Radiotherapy, Jiangsu Province Hospital of Chinese Medicine, The Affiliated Hospital of Nanjing University of Chinese Medicine, Nanjing, Jiangsu 210029, China; e Department of Gastroenterology, The First Affiliated Hospital of Nanjing Medical University, Nanjing, Jiangsu 210029, China; f Department of Gastroenterology, Huai’an Hospital Affiliated to Yangzhou University (The Fifth People’s Hospital of Huai’an), Huaian, Jiangsu 223300, China

**Keywords:** Tumor-associated macrophages, Macrophage activation, Tumor microenvironment, Molecular docking simulation

## Abstract

**Background:**

Resting tumor-associated macrophages (TAMs) are stimulated by the tumor microenvironment and can be primarily polarized into two subtypes: M1-and M2-like. M1-like TAMs promote inflammation and eradicate tumor cells, whereas M2-like TAMs suppress inflammation and facilitate tumor development. However, the mechanisms underlying phenotypic switching in these macrophages remain unclear. Therefore, we aimed to characterize the gene expression profiles of M1-like and M2-like TAMs in pan cancers.

**Methods:**

Three computational methods were used to estimate the infiltration score of TAMs in 9239 tumor samples across 31 solid cancer types, based on RNA sequencing databases. Tumor samples were divided into high- and low-score groups based on the median M1/M2 ratio. Furthermore, gene enrichment, protein interactions, and transcription factors were analyzed. Multiple pharmaco–omics profiles were used to identify potential drugs. Finally, binding between the compounds and drug targets was validated using molecular docking.

**Results:**

Among the top 100 dysregulated genes in each cancer type, 70 and 82 downregulated genes were consistently differentially expressed across most cancer types. We identified candidate drugs targeting protein phosphatase 2A (PP2A), a core protein. These included efaproxiral, hesperidin, ezetimibe, calcitriol, and linopirdine.

**Conclusions:**

This study provides a pan-cancer characterization of the TAM polarization-related gene profile. Network pharmacology and molecular docking analyses revealed five promising therapeutic agents for TAM reprogramming. Thus, our findings provide valuable insights into the enhancement of immune responses to inhibit tumor immune escape and metastasis.

## Highlights

• We characterized gene expression profiles of M1-like (immunostimulatory) and M2-like (immunosuppressive) tumor-associated macrophages using pan-cancer analysis.
• We analyzed 9239 tumor samples across 31 solid cancer types in The Cancer Genome Atlas (TCGA) online database.
• Protein phosphatase 2A (PP2A) may be a core protein involved in tumor-associated macrophage polarization according to this study.
• Drugs targeting dysregulated tumor-associated macrophage polarization-related genes were predicted using public databases.
• Efaproxiral, hesperidin, ezetimibe, calcitriol, and linopirdine are promising therapeutic agents.

## Graphical abstract

**Figure:**
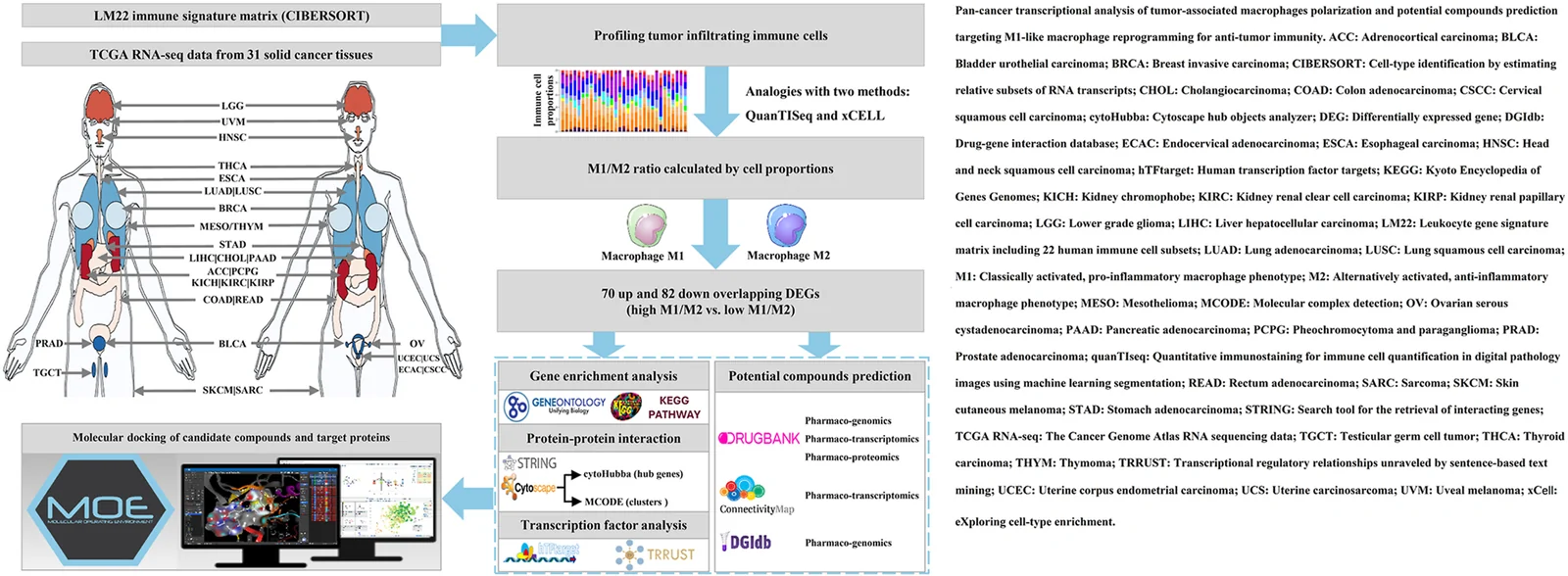

## Introduction

The mononuclear phagocyte system (MPS), a part of innate immunity, primarily comprises macrophages and their precursor cells, monocytes. Upon exposure to certain stimuli, including infection, injury, or inflammation, monocytes are recruited to the inflamed tissues and differentiate into macrophages and dendritic cells.^1^ Macrophage diversity and plasticity are hallmarks of the monocyte-macrophage lineage.^2^ Macrophages adapt to microenvironmental signals and can rapidly switch their functions by polarizing into two extreme forms *in vitro*: classically activated (M1) and alternatively activated (M2) macrophages.^3^ M1 macrophages promote inflammation and kill tumor cells. Subsets of M2 macrophages, which are mainly involved in anti-inflammatory responses and support tumor growth, include M2a, M2b, M2c, and M2d. These subtypes express distinct chemokines and chemokine receptors.^4^ Furthermore, many macrophages express a combination of M1-like and M2-like cell phenotypes.^5^, ^6^, ^7^ The tumor microenvironment (TME) modulates tumor-associated macrophages (TAMs), which mainly comprise two polarizable subtypes – M1-like TAMs and M2-like TAMs. Additionally, TAMs may reside between or outside the M1 or M2 states.^8^ The M1-like and M2-like TAM polarization balance is a highly plastic and dynamically continuous process governed by signaling pathways and transcriptional and post-transcriptional regulatory networks.^9^

The recruitment and polarization of M2-like TAMs play important roles in angiogenesis, malignant metastasis, invasion, and tumor cell dormancy.^10^ M2-like TAMs play a cancer-promoting role and serve as potential targets for overcoming resistance to anti-tumor therapy.^11^^,^^12^ Some compounds may promote the repolarization of tumor-supportive M2-like TAMs to tumor-suppressive M1-like TAMs. These compounds include monophosphoryl lipid A (MPLA), interferon (IFN) γ,^13^ lactate oxidase (LOX)^14^ and an M2-like TAM-targeting micellar nanodrug that could be triggered only in the acidic TME but not in the neutral-pH environment of healthy organs.^15^ The induction of M1-like TAMs and reprogramming of anti-inflammatory M2-like TAMs to pro-inflammatory M1-like TAMs offer a promising strategy for maximizing tumor-killing abilities and reducing pathogenicity.

Pan-cancer research integrates holistic and reductionist approaches to unify multiple analytical perspectives.^16^^,^^17^ Previous studies have focused on isolated single cancer types. In contrast, pan-cancer research explores both the heterogeneity and commonalities of cancers. Thus, the shift toward pan-cancer research provides a more comprehensive understanding of the mechanisms underlying cancer development. It simultaneously explores the universality of cancer and elucidates local mechanisms to reveal its specificity and diversity. This consequently eliminates the division and composition fallacies, which means making overly simplistic judgments about the logical relationships between the whole and its parts, in the understanding of cancer as a complex systemic disease.

The advent of single-cell RNA sequencing (scRNA-seq) technology has revealed the heterogeneity of cancer cells and the similarities in TME subtypes across different cancer types.^18^, ^19^, ^20^, ^21^, ^22^ In this study, we aimed to characterize the gene expression profiles of M1-like and M2-like TAMs in pan-cancers across 31 solid tumor types based on published RNA sequencing (RNA-seq) gene expression datasets. We further aimed to identify potential targets and pathways of TAM polarization by constructing a drug–target interaction network. By targeting macrophage polarization to shift the balance from M2-like to M1-like phenotypes, this study provides a strategy for remodeling the immunosuppressive TME, thereby restricting tumor cell survival and metastasis.

## Methods

### Data acquisition and preprocessing

We obtained the RNA-seq gene expression data of 31 solid cancers: brain lower grade glioma (LGG), uveal melanoma (UVM), head and neck squamous cell carcinoma (HNSC), thyroid carcinoma (THCA), esophageal carcinoma (ESCA), lung adenocarcinoma (LUAD), lung squamous cell carcinoma (LUSC), breast carcinoma (BRCA), mesothelioma (MESO), thymoma (THYM), stomach adenocarcinoma (STAD), liver hepatocellular carcinoma (LIHC), cholangiocarcinoma (CHOL), pancreatic adenocarcinoma (PAAD), adrenocortical carcinoma (ACC), pheochromocytoma and paraganglioma (PCPG), kidney chromophobe (KICH), kidney renal clear cell carcinoma (KIRC), kidney renal papillary cell carcinoma (KIRP), colon adenocarcinoma (COAD), rectum adenocarcinoma (READ), bladder urothelial carcinoma (BLCA), prostate adenocarcinoma (PRAD), testicular germ cell tumors (TGCTs), ovarian serous cystadenocarcinoma (OV), uterine corpus endometrial carcinoma (UCEC), uterine carcinosarcoma (UCS), endocervical adenocarcinoma (ECAC), cervical squamous cell carcinoma (CSCC), skin cutaneous melanoma (SKCM), and sarcoma (SARC) [Table 1]. The data were downloaded from The Cancer Genome Atlas (TCGA) (http://portal.gdc.cancer.gov/). The M1 and M2 macrophage compositions of the samples were calculated using three gene-based algorithms: cell-type identification by estimating relative subsets of RNA transcripts (CIBERSORT), quantitative immunostaining for immune cell quantification in digital pathology images using machine learning segmentation (quanTIseq), and eXploring cell-type enrichment (xCell).^23^, ^24^, ^25^

**Table 1:** Characterization of 31 solid cancer types in The Cancer Genome Atlas.

Solid cancer type (*n* = 31)	Grouped in M1/M2 ratio		Total samples (*n* = 9239)
	High density (≥median)	Low density (<median)	
Adrenocortical cancer	39	40	79
Bladder cancer	202	200	402
Breast cancer	536	536	1072
Cervical cancer	138	138	276
Bile duct cancer	18	18	36
Colon cancer	225	225	450
Esophageal cancer	40	40	80
Head and neck cancer	248	247	495
Kidney chromophobe	33	32	65
Kidney clear cell carcinoma	263	263	526
Kidney papillary cell carcinoma	144	143	287
Lower grade glioma	250	250	500
Liver cancer	185	184	369
Lung adenocarcinoma	255	255	510
Lung squamous cell carcinoma	248	248	496
Mesothelioma	41	40	81
Ovarian cancer	177	177	354
Pancreatic cancer	88	88	176
Pheochromocytoma & paraganglioma	88	87	175
Prostate cancer	241	240	481
Rectal cancer	82	81	163
Sarcoma	129	129	258
Melanoma	52	51	103
Stomach cancer	187	186	373
Testicular cancer	75	74	149
Thyroid cancer	248	248	496
Thymoma	60	59	119
Endometrioid cancer	268	267	535
Uterine carcinosarcoma	28	28	56
Uveal melanoma	39	38	77

M1: Classically activated, pro-inflammatory macrophage phenotype; M2: Alternatively activated, anti-inflammatory macrophage phenotype.

### Identification of gene expression profiles related to macrophage polarization in pan-cancers

Using the median ratio of M1/M2 TAMs as the cutoff point, TCGA pan-cancer samples were divided into high- and low-M1/M2 ratio groups. The differentially expressed genes (DEGs) related to macrophage polarization were obtained from RNA-seq expression profiles using the “DESeq2 (Differential gene expression analysis based on the negative binomial distribution)” R package. The cut-off criteria were adjusted *P* value (also called false discovery rate [FDR]) < 0.01 and fold change (FC) ≥ 1.5.

### Screening of potential targets using gene enrichment, protein interaction, and transcription factor analysis

Gene ontology (GO) functional analysis and Kyoto Encyclopedia of Genes Genomes (KEGG) pathways analysis^26^ against M1 and M2 macrophage-associated DEGs were performed using the GO-KEGG-Bubble tool in Hiplot (ORG) (https://hiplot.org), a comprehensive and easy-to-use web service for boosting publication-ready biomedical data visualization.^27^ FDR <0.05 indicated a statistically significant difference in enriched GO/KEGG terms.

The search tool for the retrieval of interacting genes/proteins (STRING), a database containing human proteins with core network interactions, was used to construct a protein–protein interaction (PPI) network.^28^ The interaction score was set at the highest confidence level (0.9), following the official recommendations. Key gene signatures were extracted from DEGs-based PPI complexes through molecular complex detection (MCODE) and cytoHubba (Cytoscape Hub Objects Analyzer) modules using the Cytoscape software (v3.8.0).^29^ The graph theoretic clustering algorithm, MCODE, detects core clusters representing molecular complexes in PPI networks.^30^ In this study, the Cytoscape plugin cytoHubba was used to predict hub genes in the PPI network. Eleven topological methods are provided in cytoHubba, including the Maximal Clique Centrality (MCC), Density of Maximum Neighborhood Component (DMNC), Maximum Neighborhood Component (MNC), Degree, Edge Percolated Component (EPC), and seven centralities (Bottleneck, EcCentricity, Closeness, Radiality, Betweenness, Stress, and Clustering Coefficient).^31^

Multiple interconnected factors, including transcription factor (TF) and co-factor binding, regulate macrophage differentiation, activation, and polarization.^1^ A database of human transcription factor targets (hTFtarget), which contains comprehensive TF-target regulation from chromatin immunoprecipitation sequencing (ChIP-Seq) data in cell lines, tissues, and cells, was used to identify the TF-encoding genes among macrophage polarization DEGs.^32^ Transcriptional regulatory relationships unraveled by sentence-based text mining (TRRUST) included TF-target regulatory relationships in humans from PubMed articles that describe experimental studies and provide a mode of regulation, including activation and repression.^33^ Universal Protein Knowledgebase (UniprotKB), which provides experimentally validated and article-published PPI, was used to select the final candidate drug targets.^34^

### Prediction of compounds as agonists of M1-like tumor-associated macrophage polarization or inhibitors of M2-like tumor-associated macrophage polarization in pan-cancers

We predicted the potential compounds associated with M1-like and M2-like macrophage polarization across pharmacogenomic, pharmacotranscriptomic, and pharmacoproteomic datasets in public databases. DrugBank provides pharmacotranscriptomic and pharmacoproteomic research data, including change directions, upregulated/downregulated, and increased/decreased. M1 and M2 macrophage-associated DEGs were uploaded to the Connectivity Map (CMap)^35^ on the cloud-based CLUE (CMap Legacy Umbrella Environment) software platform (https://clue.io) to identify perturbagens that elicit similar or opposing gene expression signatures. We also collected potential drugs from the drug–gene interaction database (DGIdb),^36^ a public platform that integrates resources from Drug Target Commons (DTC),^37^ Guide to Pharmacology,^38^ ChEMBL (CHEMistry BioMedicine Library),^39^ and others.

### Molecular docking of candidate compounds and drug targets

The Molecular Operating Environment (MOE) is a drug discovery software platform that includes molecular modeling, virtual screening, and stimulations.^40^ In this study, ligands (candidate compounds) were prepared in ChEMBL and minimized in MOE. Next, hydrogen atoms were added to the ligands. Protein structures of drug target genes were obtained from the Protein Data Bank (PDB).^41^ Docking was performed until the minimum energy of the drug ligand was achieved. The molecular docking scores of the generated poses of the candidate drug ligands were ranked based on the binding affinity to identify the optimal drugs.

## Results

### Differential gene expression analysis in high-vs. low-M1/M2 ratio groups in pan-cancers

**Figure 1:**
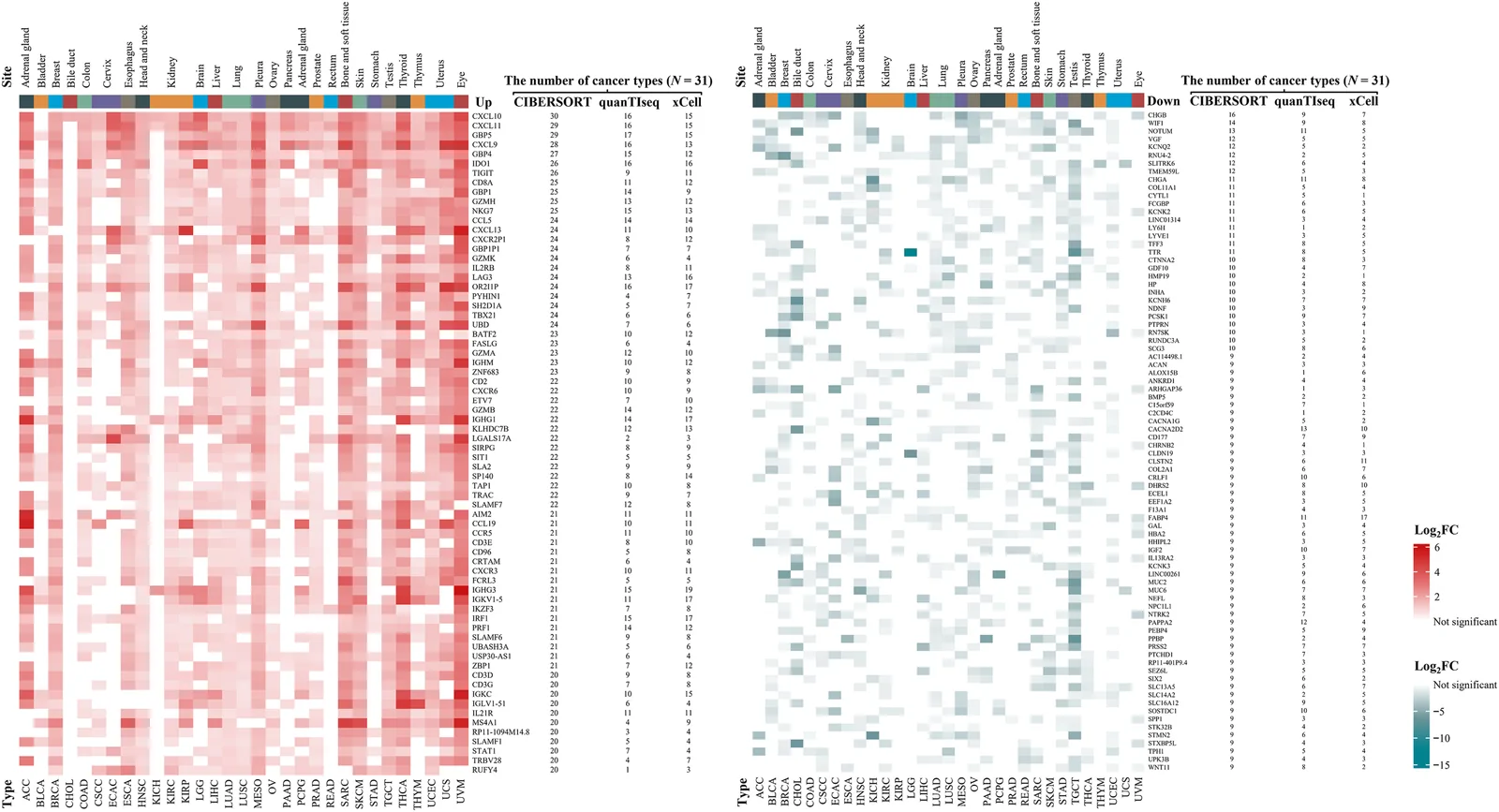
Heatmaps of the characteristic gene expression profiling of M1-and M2-like TAM polarization in 31 TCGA solid cancer-types estimated by three computational methods. The proportions of macrophages evaluated using the transcriptome-based cell-type quantification methods CIBERSORT, quanTIseq, and xCell. Higher consistency in pan-cancers was observed in the results produced via CIBERSORT. The types of cancers are listed in Table 1. ACC: Adrenocortical carcinoma; BLCA: Bladder urothelial carcinoma; CHOL: Cholangiocarcinoma; CIBERSORT: Cell-type identification by estimating relative subsets of RNA transcripts; COAD: Colon adenocarcinoma; CSCC: Cervical squamous cell carcinoma; ECAC: Endocervical adenocarcinoma; ESCA: Esophageal carcinoma; FC: Fold change; HNSC: Head and neck squamous cell carcinoma; KICH: Kidney chromophobe; KIRC: Kidney renal clear cell carcinoma; KIRP: Kidney renal papillary cell carcinoma; LGG: Lower grade glioma; LIHC: Liver hepatocellular carcinoma; LUAD: Lung adenocarcinoma; LUSC: Lung squamous cell carcinoma; M1: Classically activated, pro-inflammatory macrophage phenotype; M2: Alternatively activated, anti-inflammatory macrophage phenotype; MESO: Mesothelioma; OV: Ovarian serous cystadenocarcinoma; PAAD: Pancreatic adenocarcinoma; PCPG: Pheochromocytoma and paraganglioma; PRAD: Prostate adenocarcinoma; quanTIseq: Quantitative immunostaining for immune cell quantification in digital pathology images using machine learning segmentation; READ: Rectum adenocarcinoma; SARC: Sarcoma; SKCM: Skin cutaneous melanoma; STAD: Stomach adenocarcinoma; TCGA: The Cancer Genome Atlas; TGCT: Testicular germ cell tumor; THCA: Thyroid carcinoma; THYM: Thymoma; UCEC: Uterine corpus endometrial carcinoma; UCS: Uterine carcinosarcoma; UVM: Uveal melanoma; xCell: eXploring cell-type enrichment.

We analyzed DEGs related to M1-like and M2-like TAM polarization in 31 solid cancer types with FDR <0.01 and |log_2_FC| ≥ 0.58: LGG, UVM, HNSC, THCA, ESCA, LUAD, LUSC, BRCA, MESO, THYM, STAD, LIHC, CHOL, PAAD, ACC, PCPG, KICH, KIRC, KIRP, COAD, READ, BLCA, PRAD, TGCT, OV, UCEC, UCS, ECAC, CSCC, SKCM, and SARC. Among the top 100 DEGs in each cancer type, we identified 70 genes with upregulated expression and 82 with downregulated expression, which were consistently differentially expressed in most solid cancer types [Figure 1 and Supplementary Table 1]. Statistical analysis revealed that DEGs with downregulated expression had higher genetic heterogeneity than those with upregulated expression in the high-M1/M2 ratio *vs.* the low-M1/M2 ratio in 31 solid cancer types. For instance, the top-ranked common gene, C-X-C motif chemokine ligand 10 (*CXCL10*) is overexpressed in 30 cancer types, whereas chromogranin B (*CHGB*) expression is downregulated in 16 of the 31 TCGA solid cancers.

### Gene enrichment and pathway analyses

**Figure 2:**
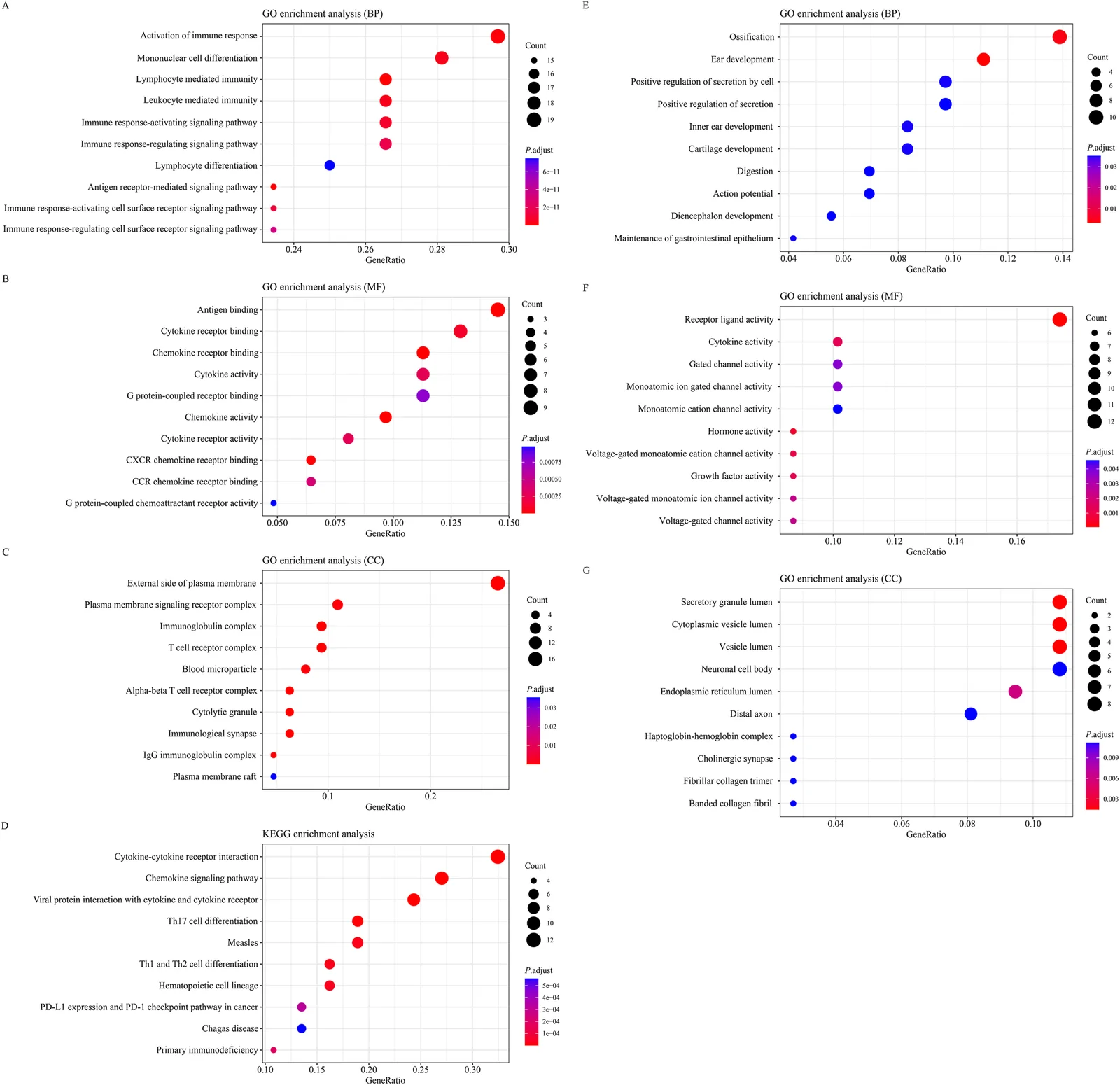
GO functional and KEGG pathway analyses of dysregulated TAM polarization-related genes. (A, B, C, and D) GO and KEGG pathway enrichment analyses of genes with upregulated expression in high-*vs.* low-M1/M2 ratio groups. (E, F, and G) GO functional analysis of genes with downregulated expression in the high-*vs.* low-M1/M2 ratio groups. No results were obtained for KEGG enrichment analysis in genes with downregulated expression. BP: Biological process; CC: Cellular component; CCR: C–C motif chemokine receptor; CXCR: C-X-C motif chemokine receptor; GO: Gene ontology; IgG: Immunoglobulin G; KEGG: Kyoto Encyclopedia of Genes and Genomes; M1: Classically activated, pro-inflammatory macrophage phenotype; M2: Alternatively activated, anti-inflammatory macrophage phenotype; MF: Molecular function; PD-1: Programmed cell death protein 1; PD-L1: Programmed cell death ligand 1; TAM: Tumor-associated macrophage; Th: T helper.

We performed gene enrichment and pathway analyses using the GO-KEGG-Bubble tool in Hiplot to elucidate the role of DEGs related to M1-like and M2-like TAM polarization in pan-cancers. The top 10 enriched GO biological processes (BPs), GO cellular components (CCs), GO molecular functions (MFs), and KEGG pathways ordered by gene count are shown in Figure 2 and listed in Supplementary Table 2.

For GO BP, up-DEGs were mainly enriched in the activation of immune response, whereas down-DEGs were primarily enriched in ossification. For the GO CCs, up-DEGs tended to be associated with the external side of the plasma membrane, whereas down-DEGs tended to be associated with the secretory granule lumen. For GO MF, several DEGs with significantly upregulated expression were enriched in antigen binding, whereas down-DEGs were enriched in receptor ligand activity.

The functional implications of the identified DEGs were further investigated using KEGG pathway analysis. The top four enriched KEGG pathways of the upregulated DEGs were cytokine–cytokine receptor interactions, chemokine signaling pathways, viral protein interactions with cytokines and cytokine receptors, and T helper 17 (Th17) cell differentiation. Downregulated DEGs were mainly enriched in cytokine–cytokine receptor interactions; however, the calculated FDR was >0.05.

### Protein–protein interaction network construction and hub gene selection

**Figure 3:**
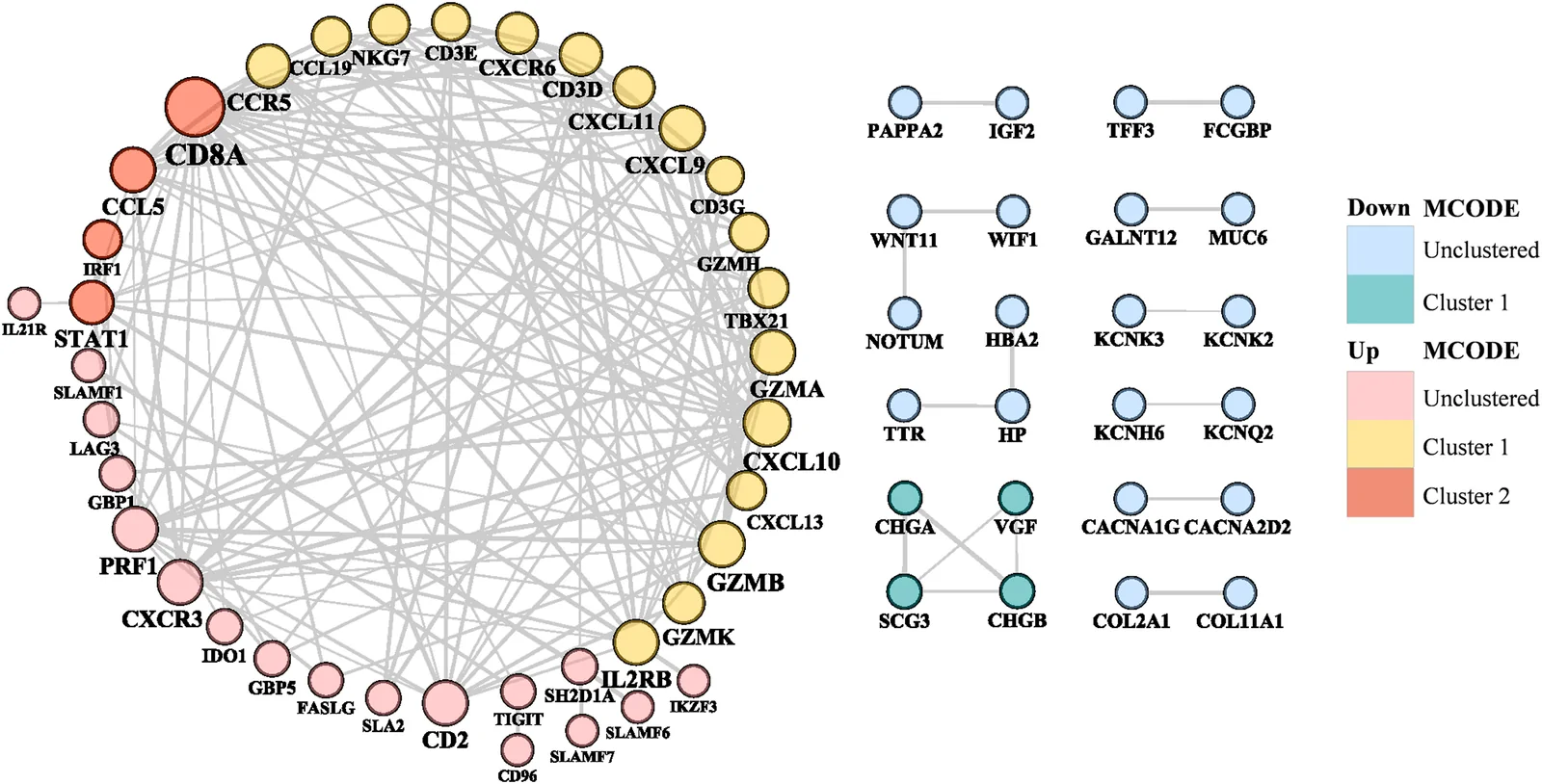
Identification of hub genes from the PPI network, as obtained from the STRING database using Cytoscape plugins, cytoHubba, and MCODE. Genes were ranked by size according to their degree scores on cytoHubba. Colors represent different MCODE clusters. cytoHubba: Cytoscape hub objects analyzer; MCODE: Molecular complex detection; PPI: Protein–protein interaction; STRING: Search tool for the retrieval of interacting genes.

A PPI network of up-DEGs was constructed, consisting of 70 nodes and 142 edges, with a mean node degree of 10. The PPI network of down-DEGs consisted of 82 nodes and 25 edges, with a mean node degree of 4. The M1-and M2-like TAM polarization-related DEGs were evaluated using the cytoHubba plugin and clustered using the MCODE plugin based on topology to locate densely connected regions with the following criteria: MCODE score >4, degree cut-off = 2, node density cut-off = 0.1, node score cut-off = 0.2, *K*-core = 2, and max depth from seed = 100. Moreover, 38 and 24 hub genes with upregulated and downregulated expression, respectively, were identified using five methods (MCC, DMNC, MNC, Degree and EPC) in cytoHubba [Supplementary Table 3]. Subsequently, hub genes were clustered in MCODE, and three significant modules were obtained from the protein network shown in Figure 3 and listed in Table 2. The seed proteins were granzyme H (GZMH) and signal transducer and activator of transcription 1 (STAT1), whose expression was upregulated. In contrast, the expression of VGF (non-acronymic, the neurosecretory protein, also known as secretogranin VII) was downregulated in high-M1/M2 ratio *vs.* low-M1/M2 ratio pan-cancers.

**Table 2:** Hub genes identified using the MCODE clustering algorithm and Cytoscape plugin cytoHubba.

M1/M2	MCODE cluster	Node	MCODE score	Degree	Betweenness centrality	Closeness centrality
Up	Cluster 1	*GZMH (seed)*	5.5714	7	0.0004	0.4512
		*CCL19*	4.4643	7	0.0006	0.4111
		*CD3E*	5.0000	6	0.0128	0.4744
		*GZMB*	4.5818	13	0.0242	0.5139
		*CXCL9*	4.9333	12	0.0335	0.5286
		*IL2RB*	5.5714	12	0.0937	0.5692
		*CXCR6*	5.0000	9	0.0224	0.5000
		*TBX21*	4.7619	8	0.0065	0.5068
		*CD3D*	5.0000	10	0.1358	0.5139
		*CXCL10*	4.9333	14	0.0846	0.5441
		*CD3G*	5.0000	6	0.0128	0.4744
		*CXCL11*	5.1667	9	0.0048	0.4302
		*GZMA*	4.9333	12	0.0209	0.5362
		*CCR5*	5.0000	11	0.0312	0.5139
		*GZMK*	4.4643	9	0.0129	0.5068
		*CXCL13*	4.4643	7	0.0006	0.4111
		*NKG7*	4.4643	8	0.0065	0.4868
	Cluster 2	*STAT1 (seed)*	3.7333	11	0.1244	0.5286
		*CCL5*	3.0128	12	0.0268	0.5286
		*CD8A*	3.1765	24	0.4087	0.7115
		*IRF1*	3.0000	7	0.0215	0.3776
Down	Cluster 1	*VGF (seed)*	4.0000	3	0.0000	0.7500
		*SCG3*	3.6667	4	0.1667	1.0000
		*CHGB*	3.6667	4	0.1667	1.0000
		*CHGA*	4.0000	3	0.0000	0.7500

String: highest confidence >0.9. Criteria: MCODE score >4, degree cut-off = 2, node score cut-off = 0.2, K-core = 2, and maximum depth from seed = 100. cytoHubba: Cytoscape hub objects analyzer; M1: Classically activated, pro-inflammatory macrophage phenotype; M2: Alternatively activated, anti-inflammatory macrophage phenotype; MCODE: Molecular complex detection.

### Transcription factor and candidate drug target screening

We queried the M1 and M2 TAM polarization-related up/downregulated DEGs of three MCODE clusters (21 up-DEGs and four down-DEGs) in the hTFtarget platform to determine whether DEGs could regulate other dysregulated genes as potential TFs at the protein level. The resulting four TFs were T-box transcription factor 21 (TBX21), STAT1, and interferon regulatory factor 1 (IRF1), which were clustered in two vital modules, and SIX homeobox 2 (SIX2), which was not significantly enriched in the clusters listed in Table 3. IRF1 can enhance the phosphorylation and activation of interferon regulatory factor 3 (IRF3) by blocking the interaction between IRF3 and its protein phosphatase 2A (PP2A) and inducing the production of IFNs after IRF3 dimerization and transfer into the nucleus.^42^ Furthermore, IRF3 activity is deactivated by the complex of PP2A and the receptor for activated C kinase 1 (RACK1), and PP2A-deficient macrophages can enhance type I IFN signaling.^43^ PP2A was ultimately selected for subsequent molecular docking of TAM polarization-related targets with the predicted compound ligands.

**Table 3:** Transcription factor prediction in dysregulated genes using the hTFtarget database.

Expression	Transcription factor	Co-regulated genes	Gene count	Dataset count
Upregulated	*TBX21*	*TAP1*, *CCL5*, *SP140*, *CRTAM*, *LAG3*, *CCR5*, *CD2*, *NKG7*, *UBASH3A*, *STAT1*, *ZBP1*, *SIT1*, *IGLV1-51*, *SLA2*, *CXCR3*, *CXCR6*, *ZNF683*, *IL2RB*, *GBP5*, *RUFY4*, *CXCR2P1*, *FASLG*, *PRF1*, *KLHDC7B*, *GBP4*, *USP30-AS1*, *AIM2*, *IRF1*, *TRAC*, *CD96*, *CCL19*, *IL21R*, *TRBV28*, *SLAMF6*, *SLAMF1*	35	3
	*IRF1*	*TAP1*, *IDO1*, *CRTAM*, *LAG3*, *STAT1*, *ZBP1*, *KLHDC7B*, *CCR5*, *GBP1P1*, *IKZF3*, *CD8A*, *ZNF683*, *GBP1*, *IL2RB*, *GBP5*, *GBP4*, *PRF1*, *CXCL11*, *AIM2*, *TRAC*, *CD96*, *USP30-AS1*, *LGALS17A*, *ETV7*, *CXCL9*, *BATF2*, *CD3E*, *CXCL10*, *UBASH3A*	29	11
	*STAT1*	*TAP1*, *IRF1*, *ETV7*, *CXCL9*, *ZBP1*, *CXCL11*, *LAG3*, *USP30-AS1*, *BATF2*, *PRF1*, *CXCL10*, *AIM2*	12	18
Downregulated	*SIX2*	*SLITRK6*, *BMP5*, *HHIPL2*, *COL11A1*, *HP*, *LYVE1*, *CRLF1*, *DHRS2*, *SCG3*, *TFF3*, *SPP1*, *RNU4-2*, *AC114498.1*, *CHGA*, *TMEM59L*, *CACNA1G*, *ANKRD1*	17	3

AIM2: Absent in melanoma 2; ANKRD1: Ankyrin repeat domain 1; BATF2: Basic leucine zipper ATF-like transcription factor 2; CCL: C-C motif chemokine ligand; CCR5: C-C chemokine receptor type 5; CD2: CD2 molecule; CD8A: CD8 subunit alpha; CD96: CD96 molecule; CHGA: Chromogranin A; CACNA1G: Calcium voltage-gated channel subunit alpha1 G; BMP5: Bone morphogenetic protein 5; CD3E: CD3e molecule; COL11A1: Collagen type XI alpha 1 chain; CRLF1: Cytokine receptor like factor 1; CRTAM: Cytotoxic and regulatory T-cell molecule; CXCL: C-X-C motif chemokine ligand; CXCR: C-X-C motif chemokine receptor; DHRS2: Dehydrogenase/reductase 2; ETV7: ETS variant transcription factor 7; FASLG: Fas ligand; PRF1: Perforin 1; GBP1P1: Guanylate binding protein 1 pseudogene 1; GBP: Guanylate binding protein; GBP5: Guanylate binding protein 5; HHIPL2: Hedgehog interacting protein like 2; HP: Haptoglobin; hTFtarget: Human transcription factor targets; IDO1: Indoleamine 2,3-dioxygenase 1; IGLV1-51: Immunoglobulin lambda variable 1-51; IKZF3: IKAROS family zinc finger 3; IL21R: Interleukin 21 receptor; IL2RB: Interleukin 2 receptor subunit beta; IRF1: Interferon regulatory factor 1; KLHDC7B: Kelch domain containing 7B; LAG3: Lymphocyte-activation gene 3; LGALS17A: Galectin 17A; LYVE1: Lymphatic vessel endothelial hyaluronan receptor 1; NKG7: Natural killer cell granule protein 7; RUFY4: RUN and FYVE domain containing 4; SCG3: Secretogranin III; SIT1: Signaling threshold regulating transmembrane adaptor 1; SIX2: SIX homeobox 2; SLAMF1: SLAM family member 1; SLA2: Src-like adaptor 2; SLAMF6: SLAM family member 6; SLITRK6: SLIT and NTRK like family member 6; SPP1: Secreted phosphoprotein 1 (osteopontin); STAT1: signal transducer and activator of transcription 1; SP140: SP140 nuclear body protein; STAT1: Signal transducer and activator of transcription 1; TAP1: Transporter associated with antigen processing 1;TBX21: T-box transcription factor 21; TFF3: Trefoil factor 3; TMEM59L: Transmembrane protein 59 like; TRAC: T cell receptor alpha constant; TRBV28: T cell receptor beta variable 28; UBASH3A: Ubiquitin associated and SH3 domain containing A; ZBP1: Z-DNA binding protein 1; ZNF683: Zinc finger protein 683.

### Potential compound prediction and molecular docking with candidate targets

**Figure 4:**
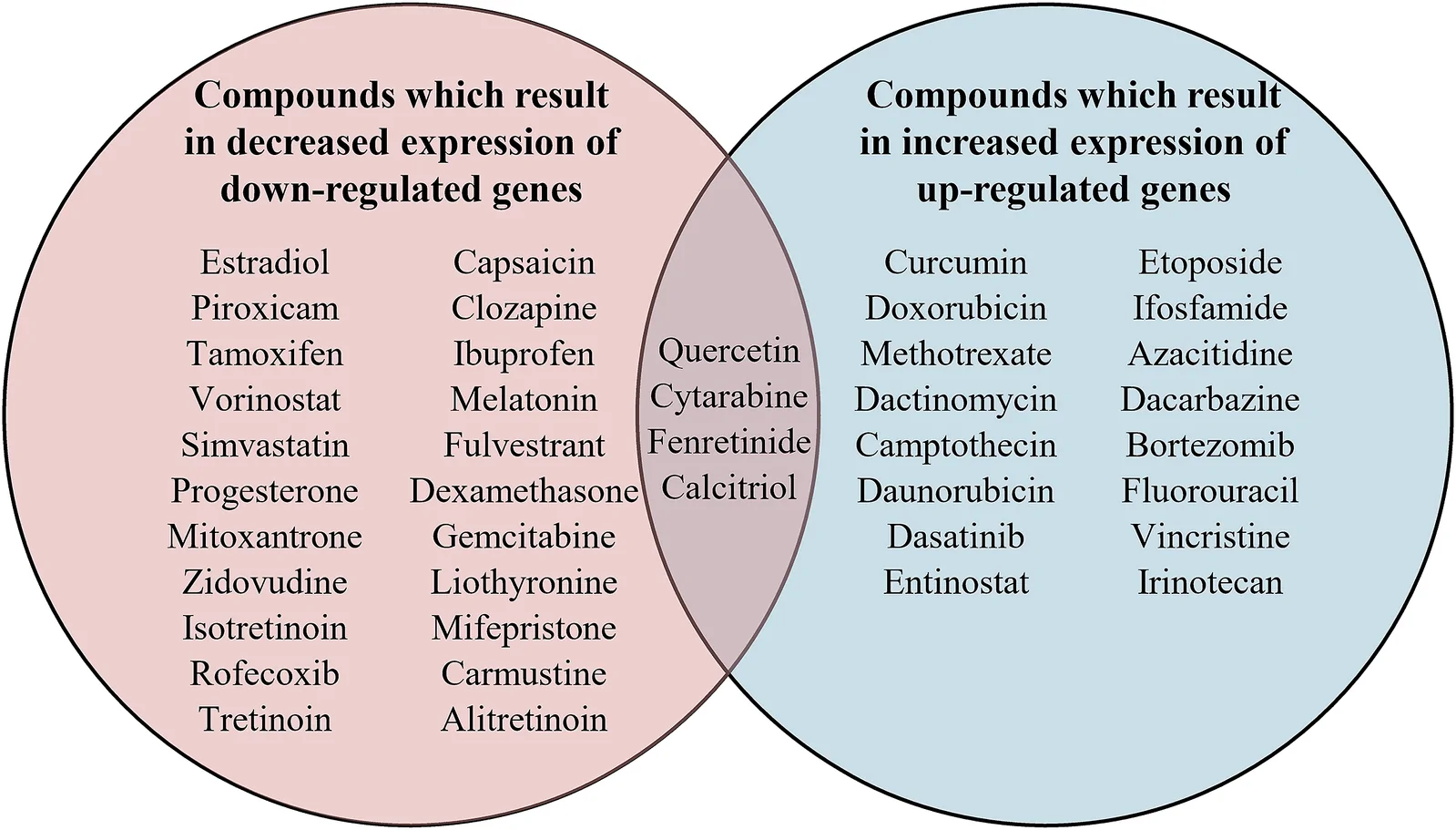
A Venn diagram of candidate compounds targeting DEGs whose expression was upregulated or downregulated in the high- and low-M1/M2 ratio groups from the DrugBank database. Four compounds were identified at the intersection: quercetin, cytarabine, fenretinide, and calcitriol. DEG: Differentially expressed genes; M1: Classically activated, pro-inflammatory macrophage phenotype; M2: Alternatively activated, anti-inflammatory macrophage phenotype.

Based on the negative log_10_*Q* value (fdr_q_nlog10) and normalized connectivity score (norm_cs), we employed CMap and the library of integrated cellular signatures (LINCS L1000) to predict potential compounds by querying the dysregulated tags of 70 up-DEGs and 82 down-DEGs separately at the transcriptomic level [Supplementary Table 4]. The negative norm_cs reflects a compound capable of generating a gene signature that exhibits a negative correlation with the input genes. The intersection compounds obtained from the CMap and pharmaco-transcriptomics datasets in DrugBank are listed in Supplementary Table 5. In total, 46 compounds were predicted to belong to the intersection of DrugBank and CMap. The compounds activating M1-like TAM polarization, blocking M2-like TAM polarization, or promoting M2 to M1 phenotype reprogramming mainly did so through inhibition of topoisomerase, histone deacetylase, dihydrofolate reductase, polar auxin transport, retinoid receptor, vitamin D receptor, estrogen receptor, nuclear factor kappa B (NF-κB) pathways. As shown in Supplementary Table 6, we also analyzed the compounds at the pharmacogenomic level (DGIdb); the intersection between DGIdb and DrugBank included five compounds: ezetimibe, hesperidin, linopirdine, phensuximide, and efaproxiral. We identified 26 compounds that decreased the expression of down-DEGs and 20 compounds that increased the expression of up-DEGs at high M1/M2 ratios *vs.* low M1/M2 ratios [Figure 4]. Four compounds were identified at the intersection: quercetin, cytarabine, fenretinide, and calcitriol.

**Figure 5:**
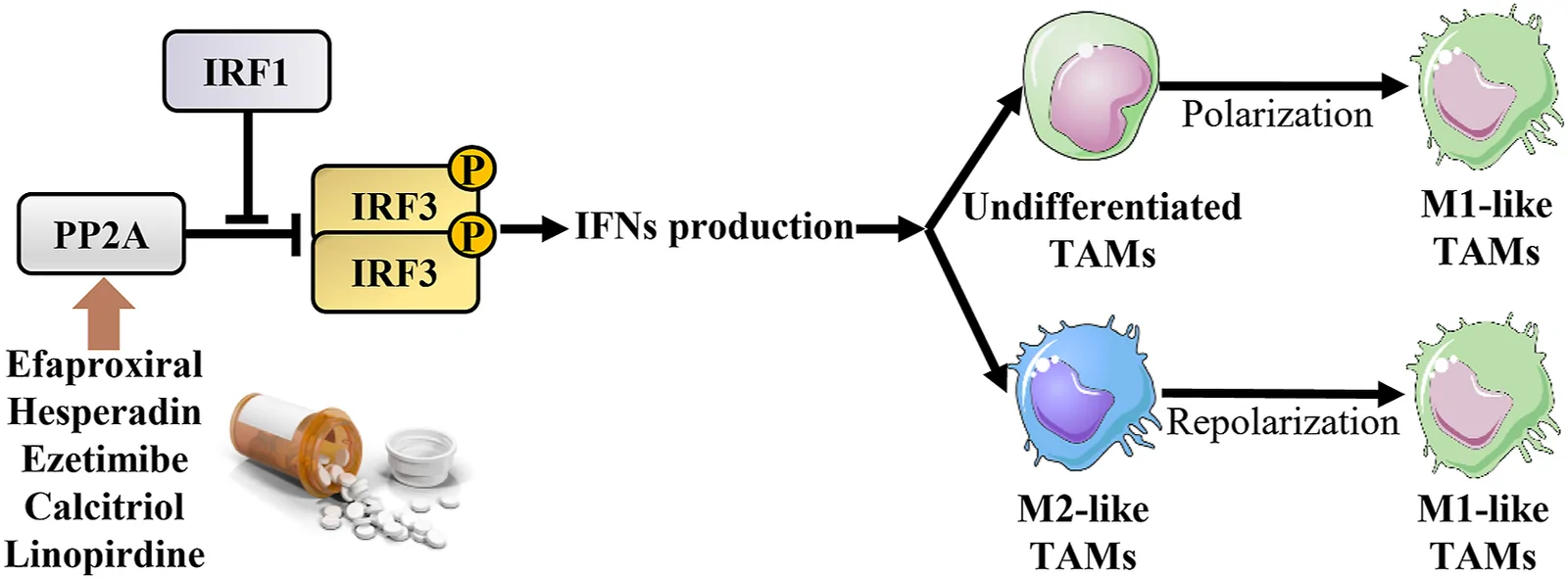
Schematic model of five compounds targeting PP2A to promote the IRF1-mediated immune response and confer anti-tumor effects by enhancing IRF3 dimerization and transfer into the nucleus. IFN: Interferon; IRF: Interferon regulatory factor; P: Phosphorylation; M1: Classically activated, pro-inflammatory macrophage phenotype; M2: Alternatively activated, anti-inflammatory macrophage phenotype; PP2A: Protein phosphatase 2A; TAM: Tumor-associated macrophage.

The 10 active ligands were subjected to molecular modeling; LB-100, a PP2A inhibitor, was used as the reference docking drug. Five of the 11 compounds were identified as potential drugs based on final molecular docking scores (MOE_S): efaproxiral (MOE_S = −6.89), hesperidin (MOE_S = −6.86), ezetimibe (MOE_S = −6.62), calcitriol (MOE_S = −6.55), and linopirdine (MOE_S = −6.41), with a cut-off score of < −6 [Table 4 and Figure 5].

**Table 4:** Results of 11 ligands docked with PP2A using MOE software.

Rank	PP2A-binding compounds	MOE_S	E_conf	E_place	E_refine
1	Efaproxiral	−6.892	−24.854	−39.327	−31.329
2	Hesperadin	−6.857	38.651	−32.951	−25.980
3	Ezetimibe	−6.624	17.719	−56.466	−33.995
4	Calcitriol	−6.553	−7.391	−69.945	−25.595
5	Linopirdine	−6.412	31.238	−45.572	−33.482
6	Fenretinide	−5.709	−48.245	−32.390	−30.974
7	LB-100	−5.622	−3.612	−57.545	−24.844
8	Quercetin	−5.438	1.293	−45.377	−29.722
9	Cytarabine	−4.912	−74.238	−54.214	−13.421
10	Phensuximide	−4.876	−28.260	−21.256	−21.027
11	Cantharidinate	−4.618	10.381	−22.819	−21.031

E_conf: Energy of the conformer; E_place: Score from the placement stage; E_refine: Score from the refinement stage; MOE: Molecular Operating Environment; MOE_S: Final molecular docking score; PP2A: Protein phosphatase 2A.

## Discussion

TAMs can be derived from circulating monocytes or tissue-resident macrophages (TRMs) that coordinate critical homeostatic and reparative functions.^44^ The significant transcriptomic diversity of monocyte/macrophage clusters has been revealed through pan-cancer analysis using single-cell sequencing technologies.^45^ Considering the tissue specificity of TAMs, we excluded some hematopoietic cancers from the analysis, including lymphoid neoplasms, diffuse large B-cell lymphoma (DLBCL), and acute myeloid leukemia-like (LAML). Rather, we focused on the 31 solid cancer types listed in Table 1. An in-depth cross-sectional comparison of the distribution of TAMs using the three algorithms demonstrated the diversity of TAMs in pan-cancers. Upregulated DEGs in the high- vs. low-M1/M2 ratio groups exhibited high expression consistency: the top-ranked gene was overexpressed in 30 out of 31 solid cancer types. However, we considered that the expression of DEGs is likely triggered by systemic chronic inflammatory responses that ultimately lead to alterations in the TME.

The activation of vital TFs regulates the skewing of macrophages toward M1- or M2-like phenotypes. For instance, STAT1, IRF9, Kruppel-like factor 6 (KLF6), and NF-κB are involved in M1-type polarization, whereas peroxisome proliferator-activated receptors (PPARs), STAT3, STAT6, IRF4, KLF4 are related to M2-type polarization.^46^ In the present study, we identified two TFs – STAT1 and IRF1 – through data analysis. The Janus kinase 1 (JAK1)/STAT1/IRF1 signaling pathway, which is activated by the binding of IFN-γ to IFN receptors, prevents mammary cancer formation by maintaining growth control.^47^ PP2A, an important phosphatase for IRF3, restricts type I IFN signaling after recruitment by RACK1 and FBXO17 (F-Box Protein 17).^41^ IRF1 may augment the phosphorylation of IRF3 by inhibiting the interaction between IRF3 and PP2A, resulting in the production of IFNs via IRF3 dimerization and nuclear import. Thus, PP2A may serve as a target for the treatment of M1-like TAM polarization.

Unknown drugs targeting PP2A were screened from candidate compounds targeting dysregulated genes in the high-and low-M1/M2 ratio groups. LB-100 was used as a positive reference docking drug to demonstrate the reliability of the docking positive drugs via conformational analysis. We identified efaproxiral, hesperidin, ezetimibe, calcitriol, and linopiridine as tumor treatments for M0-to-M1 polarization or M2-to-M1 repolarization. Efaproxiral, the compound with the best MOE score, is an allosteric modifier of hemoglobin. It can facilitate oxygen release from hemoglobin and increase tissue oxygen levels to consistently and reproducibly increase tumor oxygenation when used in combination with radiation therapy.^48^, ^49^, ^50^ The expression of JAK2 and STAT3, which can promote M2-type macrophage polarization, is significantly decreased after stimulation with hesperidin derivatives-12 (HDND-12) in M2-like TAMs.^51^

The present study has some limitations: (1) existing tools such as CIBERSORT, xCell, and quanTIseq methods measure immune cell levels but are limited to the detection of M1 and M2 types that are located at distinct opposite ends within the continuous dynamic polarization axis of TAMs. The limitation is based on M1–M2 dichotomy. Thus, future studies employing scRNA-seq are required to comprehensively characterize the molecular phenotypes of TAM subpopulations. (2) Supplemental analysis of lymphoid neoplasm DLBCL (TCGA-DLBCL) and LAMLs (TCGA-LAML), along with dysregulated gene expression profiling in M1-like *vs.* M2-like TAMs, indicated a difference between solid and hematological cancers. The present study focused on solid tumors to ensure cohort homogeneity. However, future studies are required to expand the validation to hematological malignancies and systematically assess differences in pathway activation, mutational signatures, and therapeutic vulnerabilities, which may facilitate the establishment of tissue-specific treatment strategies. (3) *In vitro* and/or *in vivo* studies are required to further validate the biological relevance of our computational findings. Although this study focused on pan-cancer bioinformatic screening to prioritize recurrently dysregulated genes, we acknowledge that functional validation remains essential. Therefore, future studies should validate the function of PP2A, along with the comparative pharmacological advantages of PP2A over upstream candidates. Furthermore, functional research is required to reveal the molecular mechanisms linking TAM polarization-related dysregulated genes in pan-cancers to their therapeutic roles.

In conclusion, pan-cancer transcriptional analysis identified DEGs in the phenotypic shifts of TAMs. Regulating the expression of these genes did not excessively increase or reduce the M1-like/M2-like TAMs ratio, which is promising for maintaining a fine-tuned balance of macrophage polarization status. Our comprehensive analysis demonstrates the expression features of TAM polarization-related genes from a pan-cancer perspective and provides new insights into M1-like TAMs reprogramming. These insights may facilitate the enhancement of immune responses to inhibit tumor immune escape and metastasis.

## Authors contribution

Xiaojing Liu and Cheng Liu: data curation, formal analysis, writing - original draft, visualization, writing - review and editing; Yuting Jin, Jing Xu, and Chunyan Xu: validation, writing - review and editing; Wei Zhu: conceptualization, investigation, supervision, writing - review and editing. All the authors have read and approved the final paper.

## Ethics statement

None.

## Data availability statement

The datasets used in this study are available from the corresponding author upon reasonable request.

## Declaration of generative AI and AI-assisted technologies in the writing process

The authors declare that generative artificial intelligence (AI) and AI assisted technologies were not used in the writing process or any other process during the preparation of this manuscript.

## Funding

None.

## Conflict of interest

The authors declare that they have no known competing financial interests or personal relationships that could have appeared to influence the work reported in this paper.

## References

[1] Kuznetsova T., Prange K.H.M., Glass C.K., de Winther M.P.J. (2020). Transcriptional and epigenetic regulation of macrophages in atherosclerosis. Nat Rev Cardiol.

[2] Shapouri-Moghaddam A., Mohammadian S., Vazini H. (2018). Macrophage plasticity, polarization, and function in health and disease. J Cell Physiol.

[3] Murray P.J., Wynn T.A. (2011). Protective and pathogenic functions of macrophage subsets. Nat Rev Immunol.

[4] Mantovani A., Sica A., Sozzani S., Allavena P., Vecchi A., Locati M. (2004). The chemokine system in diverse forms of macrophage activation and polarization. Trends Immunol.

[5] Bardi G.T., Smith M.A., Hood J.L. (2018). Melanoma exosomes promote mixed M1 and M2 macrophage polarization. Cytokine.

[6] de León U.A., Vázquez-Jiménez A., Matadamas-Guzmán M., Resendis-Antonio O. (2022). Boolean modeling reveals that cyclic attractors in macrophage polarization serve as reservoirs of states to balance external perturbations from the tumor microenvironment. Front Immunol.

[7] Zeiner P.S., Preusse C., Golebiewska A. (2019). Distribution and prognostic impact of microglia/macrophage subpopulations in gliomas. Brain Pathol.

[8] Ngambenjawong C., Gustafson H.H., Pun S.H. (2017). Progress in tumor-associated macrophage (TAM)-targeted therapeutics. Adv Drug Deliv Rev.

[9] Wang N., Liang H., Zen K. (2014). Molecular mechanisms that influence the macrophage m1-m2 polarization balance. Front Immunol.

[10] Cassetta L., Pollard J.W. (2023). A timeline of tumor-associated macrophage biology. Nat Rev Cancer.

[11] Ruffell B., Coussens L.M. (2015). Macrophages and therapeutic resistance in cancer. Cancer Cell.

[12] Wang S., Wang J., Chen Z. (2024). Targeting M2-like tumor-associated macrophages is a potential therapeutic approach to overcome antitumor drug resistance. npj Precis Oncol.

[13] Sun L., Kees T., Almeida A.S. (2021). Activating a collaborative innate-adaptive immune response to control metastasis. Cancer Cell.

[14] Liao Z.X., Fa Y.C., Kempson I.M., Tseng S.J. (2019). Repolarization of M2 to M1 macrophages triggered by lactate oxidase released from methylcellulose hydrogel. Bioconjug Chem.

[15] Xiao H., Guo Y., Li B. (2020). M2-Like tumor-associated macrophage-targeted codelivery of STAT6 inhibitor and IKKβ siRNA induces M2-to-M1 repolarization for cancer immunotherapy with low immune side effects. ACS Cent Sci.

[16] Weinstein J.N., Collisson E.A., Mills G.B. (2013). The cancer Genome atlas pan-cancer analysis project. Nat Genet.

[17] Hoadley K.A., Yau C., Hinoue T. (2018). Cell-of-Origin patterns dominate the molecular classification of 10,000 tumors from 33 types of cancer. Cell.

[18] Giraldo N.A., Sanchez-Salas R., Peske J.D. (2019). The clinical role of the TME in solid cancer. Br J Cancer.

[19] Cheng S., Li Z., Gao R. (2021). A pan-cancer single-cell transcriptional atlas of tumor infiltrating myeloid cells. Cell.

[20] Bagaev A., Kotlov N., Nomie K. (2021). Conserved pan-cancer microenvironment subtypes predict response to immunotherapy. Cancer Cell.

[21] Zheng L., Qin S., Si W. (2021). Pan-cancer single-cell landscape of tumor-infiltrating T cells. Science.

[22] Luo H., Xia X., Huang L.B. (2022). Pan-cancer single-cell analysis reveals the heterogeneity and plasticity of cancer-associated fibroblasts in the tumor microenvironment. Nat Commun.

[23] Newman A.M., Liu C.L., Green M.R. (2015). Robust enumeration of cell subsets from tissue expression profiles. Nat Methods.

[24] Finotello F., Mayer C., Plattner C. (2019). Molecular and pharmacological modulators of the tumor immune contexture revealed by deconvolution of RNA-seq data. Genome Med.

[25] Aran D., Hu Z., Butte A.J. (2017). xCell: digitally portraying the tissue cellular heterogeneity landscape. Genome Biol.

[26] Kanehisa M., Furumichi M., Sato Y., Kawashima M., Ishiguro-Watanabe M. (2023). KEGG for taxonomy-based analysis of pathways and genomes. Nucleic Acids Res.

[27] Li J., Miao B., Wang S. (2022). Hiplot: a comprehensive and easy-to-use web service for boosting publication-ready biomedical data visualization. Briefings Bioinf.

[28] Szklarczyk D., Morris J.H., Cook H. (2017). The STRING database in 2017: quality-controlled protein-protein association networks, made broadly accessible. Nucleic Acids Res.

[29] Shannon P., Markiel A., Ozier O. (2003). Cytoscape: a software environment for integrated models of biomolecular interaction networks. Genome Res.

[30] Bader G.D., Hogue C.W.V. (2003). An automated method for finding molecular complexes in large protein interaction networks. BMC Bioinf.

[31] Chin C.H., Chen S.H., Wu H.H., Ho C.W., Ko M.T., Lin C.Y. (2014). cytoHubba: identifying hub objects and sub-networks from complex interactome. BMC Syst Biol.

[32] Zhang Q., Liu W., Zhang H.M. (2020). hTFtarget: a comprehensive database for regulations of human transcription factors and their targets. Genom Proteom Bioinform.

[33] Han H., Cho J.W., Lee S. (2018). TRRUST v2: an expanded reference database of human and mouse transcriptional regulatory interactions. Nucleic Acids Res.

[34] Consortium T.U. (2022). UniProt: the universal protein Knowledgebase in 2023. Nucleic Acids Res.

[35] Subramanian A., Narayan R., Corsello S.M. (2017). A Next generation connectivity Map: L1000 platform and the first 1,000,000 profiles. Cell.

[36] Cannon M., Stevenson J., Stahl K. (2023). DGIdb 5.0: rebuilding the drug–gene interaction database for precision medicine and drug discovery platforms. Nucleic Acids Res.

[37] Tang J., Tanoli Z.U., Ravikumar B. (2018). Drug target commons: a community effort to build a consensus knowledge base for drug-target interactions. Cell Chem Biol.

[38] Harding S.D., Armstrong J.F., Faccenda E. (2024). The IUPHAR/BPS Guide to PHARMACOLOGY in 2024. Nucleic Acids Res.

[39] Gaulton A., Bellis L.J., Bento A.P. (2012). ChEMBL: a large-scale bioactivity database for drug discovery. Nucleic Acids Res.

[40] Vilar S., Cozza G., Moro S. (2008). Medicinal chemistry and the molecular operating environment (MOE): application of QSAR and molecular docking to drug discovery. Curr Top Med Chem.

[41] Berman H.M., Westbrook J., Feng Z. (2000). The protein Data Bank. Nucleic Acids Res.

[42] Wang J., Li H., Xue B. (2020). IRF1 promotes the innate immune response to viral infection by enhancing the activation of IRF3. J Virol.

[43] Long L., Deng Y., Yao F. (2014). Recruitment of phosphatase PP2A by RACK1 adaptor protein deactivates transcription factor IRF3 and limits type I interferon signaling. Immunity (Camb, Mass).

[44] Vogel A., Weichhart T. (2023). Tissue-resident macrophages - early passengers or drivers in the tumor niche?. Curr Opin Biotechnol.

[45] Wei C., Ma Y., Wang M. (2024). Tumor-associated macrophage clusters linked to immunotherapy in a pan-cancer census. npj Precis Oncol.

[46] Li H., Jiang T., Li M.Q., Zheng X.L., Zhao G.J. (2018). Transcriptional regulation of macrophages polarization by MicroRNAs. Front Immunol.

[47] Zhou F. (2009). Molecular mechanisms of IFN-gamma to up-regulate MHC class I antigen processing and presentation. Int Rev Immunol.

[48] Hou H., Khan N., Grinberg O.Y. (2007). The effects of Efaproxyn (efaproxiral) on subcutaneous RIF-1 tumor oxygenation and enhancement of radiotherapy-mediated inhibition of tumor growth in mice. Radiat Res.

[49] Stea B., Shaw E., Pintér T. (2006). Efaproxiral red blood cell concentration predicts efficacy in patients with brain metastases. Br J Cancer.

[50] Scott C., Suh J., Stea B., Nabid A., Hackman J. (2007). Improved survival, quality of life, and quality-adjusted survival in breast cancer patients treated with efaproxiral (Efaproxyn) plus whole-brain radiation therapy for brain metastases. Am J Clin Oncol.

[51] Sharifnia M., Eftekhari Z., Mortazavi P. (2024). Niosomal hesperidin attenuates the M1/M2-macrophage polarization-based hepatotoxicity followed chlorpyrifos -induced toxicities in mice. Pestic Biochem Physiol.

